# A Novel Androgen-Induced lncRNA FAM83H-AS1 Promotes Prostate Cancer Progression *via* the miR-15a/CCNE2 Axis

**DOI:** 10.3389/fonc.2020.620306

**Published:** 2021-02-04

**Authors:** Bo Liu, Duocheng Qian, Weidong Zhou, Huiyang Jiang, Zhendong Xiang, Denglong Wu

**Affiliations:** ^1^ Department of Urology, Tongji Hospital, Tongji University of Medicine, Shanghai, China; ^2^ Department of Urology, Shanghai Fourth People’s Hospital affiliated to Tongji University School of Medicine, Shanghai, China

**Keywords:** prosate cancer, long non-coding RNA, FAM83H-AS1, CCNE2, proliferation, migration

## Abstract

Prostate cancer (PCa) is one of the most common types of tumors among males worldwide. However, the roles of long noncoding RNAs (lncRNAs) in PCa remain unclear. This study shows that lncRNA FAM83H-AS1 is upregulated in prostate adenocarcinoma, bladder urothelial carcinoma, and kidney renal papillary cell carcinoma samples. Androgen receptor (AR) signaling plays the most important role in PCa tumorigenesis and development. In this study, the results validate that AR signaling is involved in upregulating FAM83H-AS1 expression in PCa cells. Loss-of-function assays demonstrate that FAM83H-AS1 acts as an oncogene in PCa by modulating cell proliferation, cell cycle, and migration. Bioinformatics analysis demonstrates that FAM83H-AS1 is remarkably related to the regulation of the cell cycle and DNA replication through affecting multiple regulators related to these pathways, such as CCNE2. Mechanically, we found that FAM83H-AS1 plays its roles through sponging miR-15a to promote CCNE2 expression. These findings indicate that FAM83H-AS1 is a novel diagnostic and therapeutic marker for PCa.

## Introduction

As one of the most common kinds of tumors (1), the mechanism related to the tumorigenesis and development of prostate cancer (PCa) remains unclear. Most recently, long noncoding RNAs (lncRNAs) were revealed to have a key role in PCa by affecting cell proliferation, metastasis, apoptosis, autophagy, and chemoresistance (2, 3). Mechanically, lncRNAs could regulate multiple PCa-related oncogenes and tumor suppressors (2–6). For example, lncRNA HOTAIR promotes castration-resistant PCa progression through enhancing the transcriptional activity of androgen receptors (ARs) (6). The lncRNA ARLNC1 promotes PCa proliferation through enhancing AR signaling *via* RNA–RNA interaction (2). SChLAP1 promotes PCa migration and invasion through suppressing SWI/SNF chromatin-modifying complex (3). Exploring the molecular functions of lncRNAs may help us to understand the pathogenesis of PCa.

FAM83H-AS1 is an lncRNA related to cancer progression regulation (7–11). FAM83H-AS1 is reported to be overexpressed in hepatocellular carcinoma (7), colon cancer (8), lung cancer (9), gastric cancer (10), and bladder cancer (11). FAM83H-AS1 is reported to be an oncogene through affecting cell proliferation, migration, radioresistance, and proliferation in cancers (12, 13). For example, FAM83H-AS1 is upregulated and correlated to poor prognosis of glioma (12). Knockdown of this lncRNA induced glioma cell cycle arrest and apoptosis *via* epigenetically regulating CDKN1A (p21) (12). In ovarian cancer, Dou et al. find that FAM83H-AS1 promotes tumor radioresistance and progression *via* HuR protein (13). However, the clinical importance and functions of FAM83H-AS1 in prostate cancer are still unknown.

In this study, we performed multi-institutional analysis to identify differently expressed lncRNAs in PCa by using TCGA and GEO data sets. FAM83H-AS1 is reported to be overexpressed in PCa. Bioinformatics analysis and experimental methods were both applied to investigate the roles of FAM83H-AS1 in PCa. Our findings strongly indicate that FAM83H-AS1 is related to PCa progression and is a multifunctional and promising biomarker

## Material and Methods

### Public Data Set Analysis

The differently expressed mRNAs and lncRNAs in urinary cancers were downloaded from GEPIA data sets. The GSE513217 data set was used to confirm the upregulation of FAM83H-AS1 in PCa. A public ChIP-seq data set GSE55062 was used to confirm that FAM83H-AS1 is a direct target of AR.

The online software STRING (https://string-db.org/cgi/input?sessionId=bqhwhSS4047Q&input_page_show_search=on) was used to construct the protein–protein interaction (PPI) network.

### Tissue Collection

Eight normal prostate tissues and 20 PCa samples were acquired from Tongji Hospital between January 2001 and December 2013, and this was approved by the ethics committee of Tongji University. Written informed consent was acquired from all participants.

### Cell Culture, Androgen Treatment, and Transfection

All cells were purchased from the ATCC and cultured in an RPMI1640 medium with 10% FBS (GIBCO) at 37°C with 5% CO_2_.

siRNAs were obtained from GenePharma (GenePharma) and transfected into PCa cells using a RNAiMAX reagent (Invitrogen). The siRNAs are listed in **Table S1**. All of these assays were conducted according to the supplier’s instructions. The sequences of siRNAs were as follows: si#FAM83H-AS1-1: 5′-CCGGTGGCCTCTTGTTATT-3′, si#FAM83H-AS1-2: 5′- CCTCTTGTTATTGACCCTT-3′, and si-AR: 5′- CCGAGGAGCUUUCCAGAAU-3′.

### qRT-PCR Analysis

RNA was isolated using Trizol reagent (Sangon Inc.). Reverse transcription was applied using a PrimeScript™ RT reagent kit (Takara). qRT-PCR was conducted with SYBR green PCR Master Mix (TOYOBO) with the ABI 7500 system. Primers are listed in **Table S1**. Relative levels of genes were determined using the 2^-ΔΔCt^ method. All of these assays were conducted based on the supplier’s instructions.

### Chromatin Immunoprecipitation (ChIP) Assay

ChIP was conducted according to a previous report (14).

### Cell Proliferation Assay

CCK-8 (Dojindo) was applied to detect cell proliferation with or without transfection, and 2000 transfected cells were cultured in 96-well plates and detected each day with a microplate reader (Bio-Tek) based on the supplier’s instructions.

### Cell Cycle Assay

Transfected cells were fixed in 0.03% triton X-100 at room temperature for 5 min and were stained with propidium oxide with a FACScalibur flow cytometer (BD) and analyzed with ModFit software (Verity Software House) following the supplier’s instructions.

### Transwell Assay

For this, 2×10^5^ cells in 100 µL FBS-free medium were transferred to the top chamber of 8-μm culture inserts (Corning) coated with or without 50 µg matrigel matrix dilution (BD, Bedford, MA, USA). Twenty percent FBS-DMEM was added to the lower chamber of the culture inserts. After 24 h, these inserts were treated with methanol for 10 min and stained by DAPI for 10 min. A Leica DMI4000B microscope (Leica Microsystems, Heidelberg, Germany) was utilized for counting migrated and invaded cells in three random fields (×20).

### Western Blotting Analysis

Western blotting was conducted according to a previous report (15) using antibodies against CCNE2 (ab226972, Abcam) and GAPDH (ab9485, Abcam).

### Dual-Luciferase Reporter Assay

Relative luciferase activity was determined with the Dual‐Luciferase^®^ Reporter Assay System based on supplier’s instructions (Promega).

### Statistical Analysis

All results are shown as mean ± SEM. By using GraphPad Prism software, we determined the statistical analyses using *T*-test. A *p* < 0.05 was considered statistical significance.

## Results

### Screening of Differently Expressed lncRNAs in Urinary Cancers

**Figure 1:**
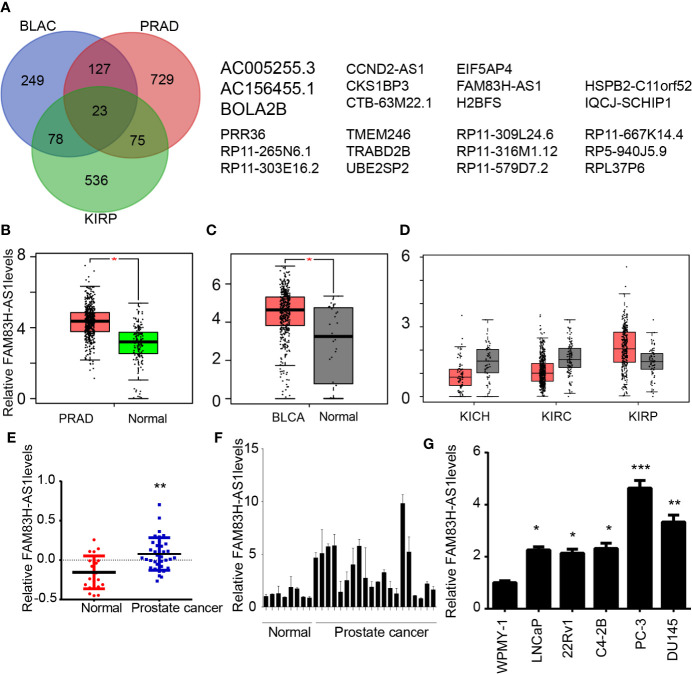
FAM83H-AS1 was dysregulated in PCa, bladder cancer, and kidney cancer. **(A)** Screening of differently expressed lncRNAs in PRAD, BLAC, and kidney cancer. **(B–D)** FAM83H-AS1 was enhanced in PRAD **(B)**, BLAC **(C)**, and KIRP **(D)** by analyzing GEPIA databases. **(E)** FAM83H-AS1 was enhanced in PRAD by analyzing the GSE5132 database. **(F)** qRT-PCR analysis of FAM83H-AS1 expressions in 20 PCa samples and 8 normal tissues. **(G)** qRT-PCR analysis of FAM83H-AS1 expressions in cell lines. *p < 0.05, **p < 0.01; ***p < 0.001.

Bladder, kidney, and prostate cancers are the most common cancers in the urinary system. To determine the mechanisms involved in regulating tumor development, we screened differently expressed lncRNAs in these cancers. A total of 687 dysregulated lncRNAs in prostate adenocarcinoma (PRAD), 546 dysregulated lncRNAs in bladder urothelial carcinoma (BLAC), and 368 dysregulated lncRNAs in kidney renal papillary cell carcinoma (KIRP) were identified by using GEPIA (16) data sets (**Figure 1A**).

By integrating the analysis of these lncRNAs, 24 lncRNAs were found to be differently expressed in PRAD, BLAC, and KIRP, indicating that they play regulatory roles in the progression of these cancers (**Figure 1A**). Among these genes, the present study focused on the novel lncRNA FAM83H-AS1, which is related to the development of multiple human cancers and whose functions in prostate cancer remained largely unknown.

### FAM83H-AS1 Is Upregulated in PCa

As presented in **Figure 1**, the results reveal that FAM83H-AS1 is upregulated in PRAD (**Figure 1B**) and BLAC (**Figure 1C**). We also analyzed the expression level of FAM83H-AS1 in kidney chromophobe (KICH), kidney renal clear cell carcinoma (KIRC), and KIRP. Our results show that FAM83H-AS1 is upregulated in KIRP; however, it is downregulated in KIRC and KICH samples compared to normal samples. Furthermore, the GEO data set GSE5132 (17) was used to validate the upregulation of FAM83H-AS1 in PCa. The result demonstrates that this lncRNA is indeed upregulated in PCa compared to normal prostate tissues (**Figure 1E**).

To validate the expression profile of FAM83H-AS1 in public data sets, we detected FAM83H-AS1 levels in PCa samples. The expression of FAM83H-AS1 in 20 PCa was higher than that in 8 normal prostate tissues, suggesting the potential important role of FAM83H-AS1 in PCa (**Figure 1F**). We detected the expression of this lncRNA in PCa-related cell lines with RT-PCR. The results indicate that FAM83H-AS1 is upregulated in PCa cell lines compared to normal prostate WPMY-1 cells (**Figure 1G**).

### FAM83H-AS1 Is a Direct Target of AR

**Figure 2:**
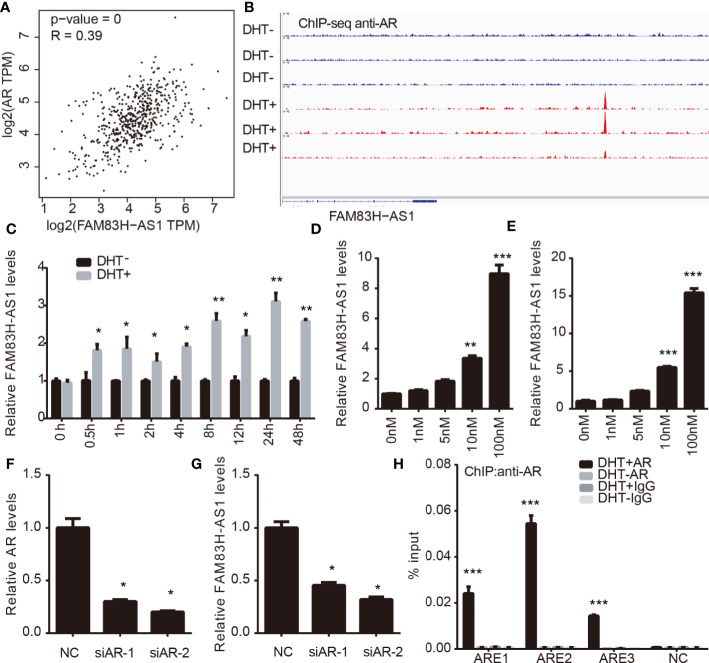
FAM83H-AS1 is direct target of AR. **(A)** Co-expression analysis indicates the significantly positively correlation between FAM83H-AS1 and AR levels inPCa. **(B)** AR peaks in FAM83H-AS1 loci significantly increased after DHT treatment compared with the control group by analyzing GSE55062. **(C)** FAM83H-AS1 expression was detected withqRT-PCR after DHT treatment in LNCaP cells. **(D, E)** qRT-PCR analysis showed FAM83H-AS1 was significantly induced under DHT treatment in a dose-dependentmanner. **(F, G)** qRT-PCR analysis was used to detect FAM83H-AS1 expression after knockdown of AR in PCa cells. **(H)** ChIP-PCR assay revealed AR was recruitedto the AREs of FAM83H-AS1 in LNCaP cells after DHT treatment for 4 h. *p < 0.05, **p < 0.01, ***p < 0.001.

AR has a key role in PCa by regulating downstream proteins, lncRNAs, and miRNAs. By conducting coexpression analysis of AR in PCa using a TCGA data set, we identified 2987 potential AR-regulating genes with absolute Pearson correlation coefficient ≥ 0.3, including 254 lncRNAs and 2733 mRNAs. Very interestingly, we observed that FAM83H-AS1 was significantly positively correlated to the expression levels of AR (*p* < 0.001, *R* = 0.39) (**Figure 2A**). To further validate these results, we analyzed public ChIP-seq data sets involved in AR, including GSE55062 (18, 19). As shown in **Figure 2**, we found AR peaks in FAM83H-AS1 loci significantly increased after DHT treatment compared to the control group by analyzing GSE55062 (**Figure 2B**).

We next detected the expression of FAM83H-AS1 in LNCaP cells after treating with DHT. We found that, in LNCaP cells, FAM83H-AS1 upregulation increased after DHT treatment (**Figure 2C**). Then, a dose-dependent DHT stimulation assay indicated that FAM83H-AS1 was significantly induced in both LNCaP and LNCaP-AI cells (**Figures 2D, E**). Moreover, we revealed that AR knockdown remarkably decreased FAM83H-AS1 levels in LNCaP and LNCaP-AI cells (**Figures 2F, G**). Finally, ChIP-PCR assay revealed that AR was remarkably enriched in the AREs of FAM83H-AS1 after treating with DHT for 4 h compared with control (**Figure 2H**). The potential androgen response elements (AREs) around FAM83H-AS1 transcription start sites were predicted according to the Genomatix database. Collectively, these findings show AR directly regulates FAM83H-AS1.

### Function Enrichment of FAM83H-AS1

We performed function enrichment analysis to reveal the potential roles of FAM83H-AS1 using its coexpressing genes according to Guttman et al’s report (18). By using the GEPIA database (http://gepia.cancer-pku.cn/), the top 200 genes were selected as the potential targets of FAM83H-AS1 in PCa.

**Figure 3:**
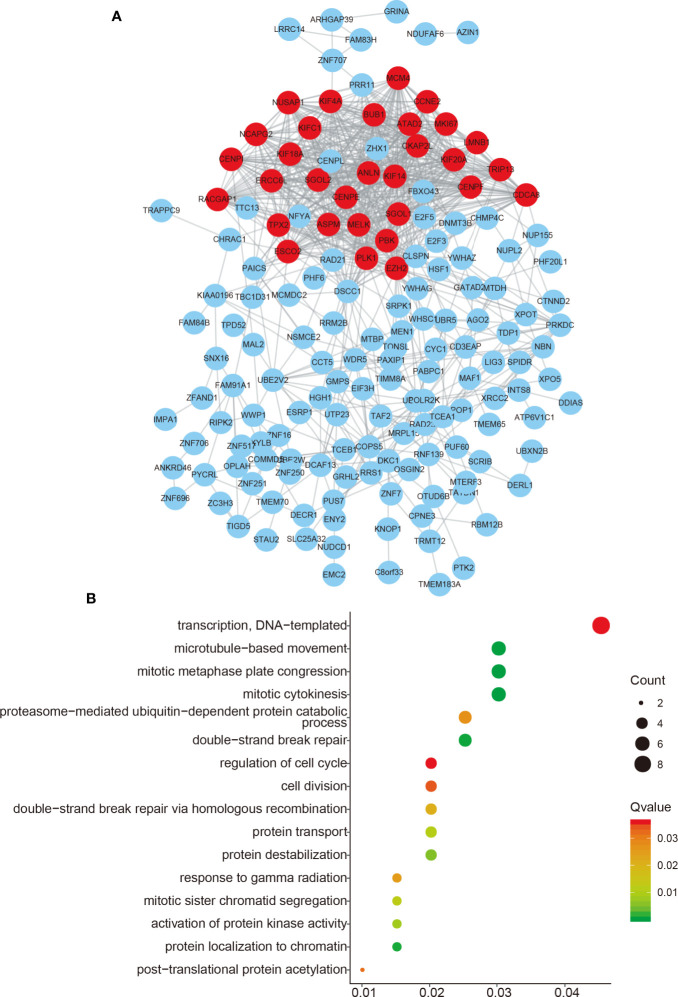
Functional enrichment of FAM83H-AS1. **(A)** The PPI network analysis of FAM83H-AS1 co-expressing genes in PCa. **(B)** Bioinformatics analysis of FAM83H-AS1 in PCa using the DAVID system.

Go analysis revealed that FAM83H-AS1 was enriched in mitotic cytokinesis, mitotic metaphase plate congression, microtubule-based movement, double-strand break repair, transcription, cell cycle, cytokinesis, telomere capping, and DNA replication, demonstrating that FAM83H-AS1 may play a crucial role in promoting PCa proliferation (**Figure 3A**). The PPI network was further used to reveal the interaction among 200 genes (**Figure 3B**).

### Knockdown of FAM83H-AS1 Suppressed PCa Cell Proliferation and Migration

**Figure 4:**
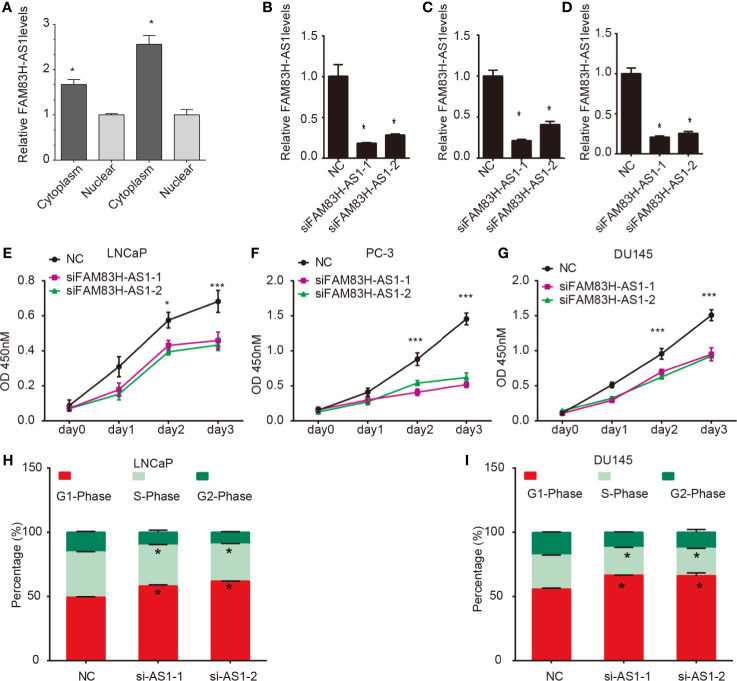
Silencing of FAM83H-AS1 suppressed PCa cell proliferation. **(A)** FAM83H-AS1 was located in cytoplasm. **(B–D)** The silencing efficiency of FAM83H-AS1 in LNCaP **(B)**, PC-3 **(C)** and DU145 **(D)** were detected using qRT-PCR analysis. **(E–G)** FAM83H-AS1 knockdown suppressed the viability rate compared to control group in LNCaP **(E)**, PC-3 **(F)**, and DU145 **(G)**. **(H, I)** Flow cytometry assay showed the percentage of G1 phase and S phase after knockdown of FAM83H-AS1 in LNCaP **(G, H)** and DU145 **(I, J)** cells. *p < 0.05; ***p < 0.001.

We first determined the subcellular location of LINC00304 in LNCaP and DU-145 cells and showed that LINC00304 was located in cytoplasm (**Figure 4A**). Next, we used siRNAs to evaluate the roles of FAM83H-AS1 in PCa. The knockdown efficiency is shown in **Figure 4** (**Figures 4B–D**). Then, the CCK-8 assay was employed, and the results show FAM83H-AS1 knockdown significantly inhibited the proliferation rate in LNCaP (**Figure 4E**), PC-3 (**Figure 4F**), and DU145 (**Figure 4G**). In addition, a flow cytometry assay showed knockdown of FAM83H-AS1 contributed to the increase of cells in the G1 phase and the decreased of cells in S phase in LNCaP (**Figure 4H**, **Figure S1A**) and DU145 (**Figure 4I**, **Figure S1B**) cells. These findings indicate that FAM83H-AS1 has a similar role in both AR-positive and AR-negative PCa cells.

**Figure 5:**
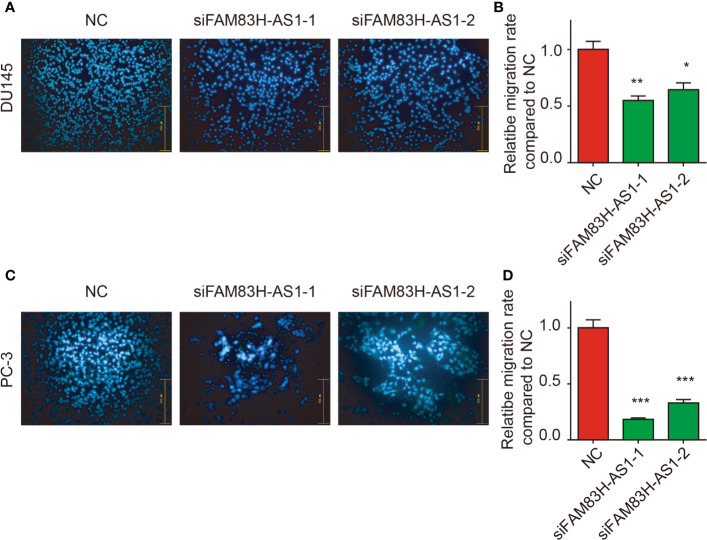
Silencing of FAM83H-AS1 suppressed cell metastasis of PCa cells. **(A, B)** Transwell assay revealed silence of FAM83H-AS1 significantly inhibited the ability of cell migration in PC-3 cells. **(C, D)** Transwell assay revealed silence of FAM83H-AS1 significantly inhibited the ability of cell migration in DU145 cells. *p < 0.05, **p < 0.01, ***p < 0.001.

Moreover, a transwell assay showed silencing of FAM83H-AS1 suppressed PC-3 (**Figures 5A, B**) and DU145 cell migration (**Figures 5C, D**).

### FAM83H-AS1 Enhanced CCNE2 Expression *via* miR-15a

Co-expression analysis indicates that FAM83H-AS1 was significantly correlated to CCNE2 expression in PCa. Moreover, bioinformatics analysis indicates that FAM83H-AS1 has a potential effect on the miR-15a/CCNE2 axis. miR-15a has been demonstrated to suppress the progression of multiple cancers, including PCa. CCNE2 is involved in regulating the cancer cycle, apoptosis, and metastasis.

**Figure 6:**
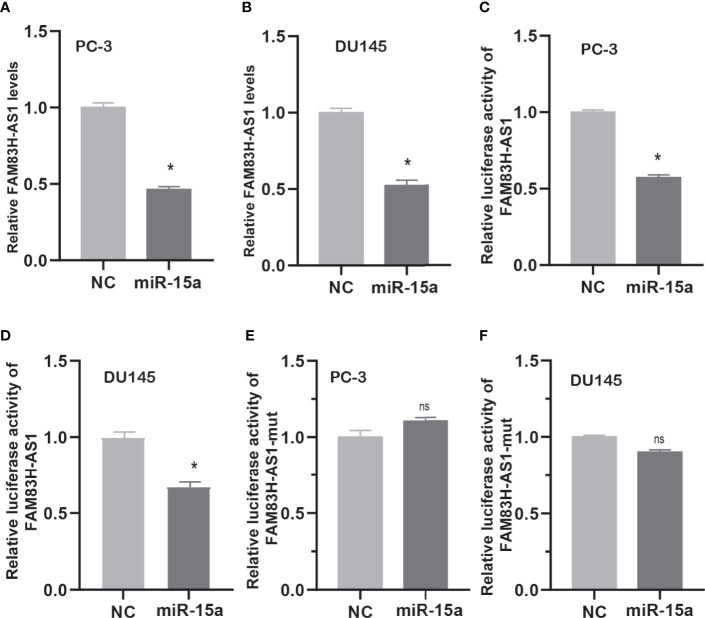
FAM83H-AS1 sponged miR-15a in PCa cells. **(A, B)** qRT-PCR analysis was used to detect FAM83H-AS1 expression after overexpression of miR-15a in PC-3 **(A)** and DU145 **(B)**. **(C, D)** miR-15a mimic reduced the luciferase activity of the FAM83H-AS1 luciferase reporter vector in PC-3 **(C)** and DU145 **(D)**. **(E, F)** miR-15a mimic did not affect the luciferase activity of the FAM83H-AS1 mutant luciferase reporter vector in PC-3 **(E)** and DU145 **(F)**. *p < 0.05. nsn not significant.

Then, we detected FAM83H-AS1 expression after overexpressing miR-15a in PCa cells. As present in **Figure 8**, we find the RNA levels of FAM83H-AS1 were suppressed after transfecting miR-15a in PC-3 (**Figure 6A**) and DU145 (**Figure 6B**) cells. After cotransfecting FAM83H-AS1 wild-type or mutant luciferase reporter plasmids with miR-15a in PCa cells, we found the relative luciferase activity of the FAM83H-AS1 wild-type reporter (**Figures 6C, D**), not FAM83H-AS1 mutant reporter (**Figures 6E, F**), was suppressed after overexpressing miR-15a in both PC-3 and DU145.

**Figure 7:**
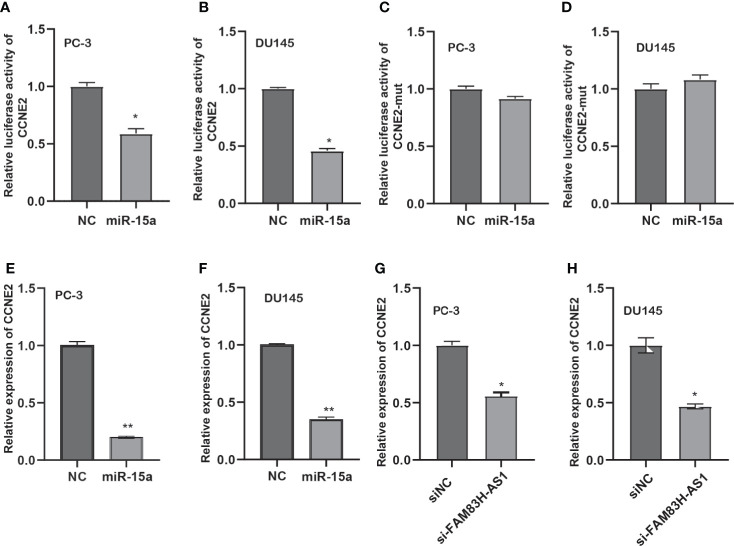
FAM83H-AS1 upregulated CCNE2 *via* miR-15a in PCa. **(A, B)** An miR-15a mimic suppressed the luciferase activity of the CCNE2 vector in PC-3 **(A)** and DU145 **(B)**. **(C, D)** An miR-15a mimic did not affect the luciferase activity of the CCNE2 mutant vector in PC-3 **(C)** and DU145 **(D)**. **(E, F)** qRT-PCR analysis was used to detect CCNE2 expression after overexpression of miR-15a in PC-3 **(E)** and DU145 **(F)**. **(G, H)** qRT-PCR analysis was applied to detect CCNE2 expression after knockdown of FAM83H-AS1 in PC-3 **(G)** and DU145 **(H)**. *p < 0.05, **p < 0.01.

Next, we detected whether FAM83H-AS1 modulates CCNE2 *via* miR-15a. We found that miR-15a suppressed the luciferase activity of CCNE2 wild type, but not mutant CCNE2 (**Figures 7A–D**). Moreover, miR-15a decreased the RNA and protein levels of CCNE2 in PCa cells (**Figures 7E–G**). In addition, our results report that silencing of FAM83H-AS1 suppressed CCNE2 expression in PC-3 and DU145 cells (**Figures 7H–J**).

### FAM83H-AS1 Enhanced PCa Cell Proliferation and Migration Through CCNE2

**Figure 8:**
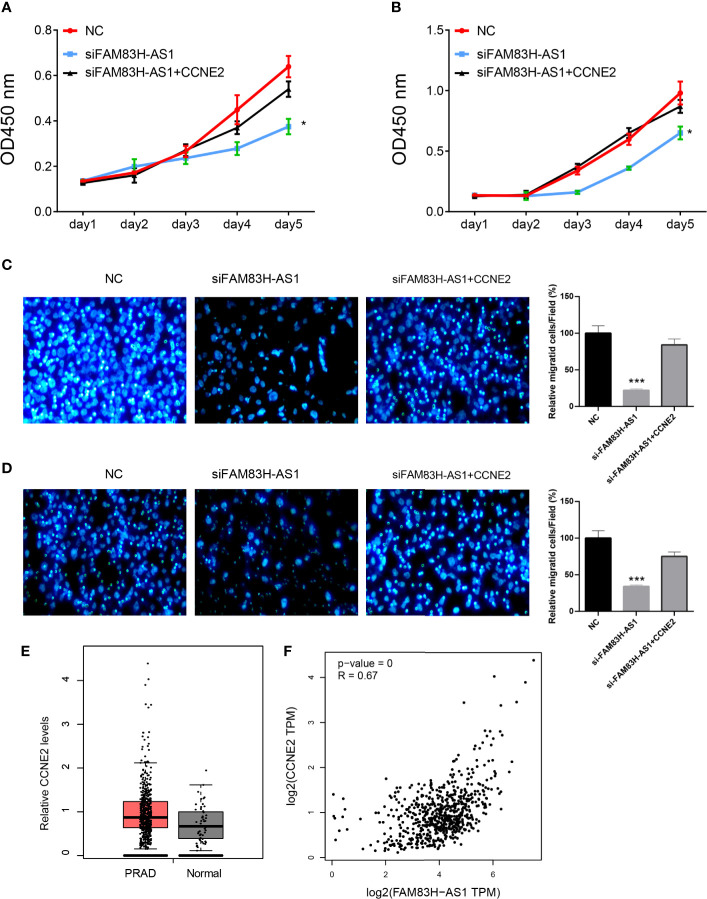
FAM83H-AS1 enhanced PCa progression *via* CCNE2. **(A, B)** CCNE2 rescued the proliferation suppression after knockdown of FAM83H-AS1 in both PC-3 **(A)** and DU145 **(B)** cells. **(C, D)** Overexpression of CCNE2 rescued the migration suppression after knockdown of FAM83H-AS1 in both PC-3 **(A)** and DU145 **(B)** cells. **(E)** The results show that CCNE2 was overexpressed in PCa samples compared to normal prostate tissues. **(F)** The results show that FAMB3H-AS1 significantly correlated to CCNE2 in PCa samples using GEPIA database. *p < 0.05; ***p < 0.001.

To test whether FAM83H-AS1 promoted the progression of PCa though CCNE2, we conducted recuse experiments and found cotransfection of siFAM83H-AS1 and CCNE2 promoted PCa cell proliferation and migration compared to transfection of siFAM83H-AS1 (**Figures 8A–D**).

Finally, we analyzed the expression levels of CCNE2 in PCa. The results show that CCNE2 was overexpressed in PCa samples compared to normal prostate tissues (**Figure 8E**). We also analyzed the associations between CCNE2 and FAMB3H-AS1. The results show FAMB3H-AS1 significantly correlates to CCNE2 in PCa samples using GEPIA database (**Figure 8F**).

## Discussion

In this study, we identified that a novel lncRNA, FAM83H-AS1, plays a crucial role in PCa. We show that FAM83H-AS1 is overexpressed in PRAD, BLAC, and KIRP samples. Moreover, the results validate that AR signaling is involved in upregulating FAM83H-AS1 expression in PCa cells. Loss-of-function assays demonstrate that FAM83H-AS1 has a oncogenetic role in PCa by modulating cell proliferation, apoptosis, and migration. Mechanically, we found that FAM83H-AS1 played its roles though sponging miR-15a to promote CCNE2 expression. These findings showed FAM83H-AS1 is a potential diagnostic and therapeutic marker for PCa. Very interestingly, an independent study demonstrates that FAM83H-AS1 was involved in regulating tumorigenesis in bladder cancer, supporting that FAM83H-AS1 is a key regulator in urinary cancers. LncRNAs have a key role in PCa progression. For example, CCAT1 interacts with miR-28-5p to promote PCa cell proliferation (20). PAX5-induced FOXP4-AS1 sponged miR-3184-5p to induce PCa growth (21). HORAS5 is related to castration-resistant PCa through modulating AR signaling (22). The present study focuses on exploring the functions of a novel lncRNA FAM83H-AS1 in PCa. FAM83H-AS1 is widely reported as an oncogene in multiple cancer types (7, 10, 12, 23). This lncRNA could affect cancer cell growth and metastasis in cancer cells. This study shows that FAM83H-AS1 is an AR-regulating lncRNA. Co-expression analysis reveals that FAM83H-AS1 significantly correlates to the expression of AR. An RT-PCR assay shows that FAM83H-AS1 expression was induced after DHT treatment. A ChIP-PCR assay and ChIP-seq show that AR protein is recruited to the AREs of FAM83H-AS1 after DHT treatment compared with the control. These findings show AR directly regulates FAM83H-AS1. FAM83H-AS1 knockdown reduces PCa cell proliferation but promotes cell cycle arrest and apoptosis. Distant metastases remain a challenge in the treatment of PCa. Therefore, we focus on exploring the effects of FAM83H-AS1 on PCa metastasis. This study demonstrates that FAM83H-AS1 silencing suppresses PC-3 and DU145 migration.

Over the past decades, CeRNA regulation of lncRNAs is demonstrated to be a key mechanism driving cancer progression. For example, ABHD11-AS1 modulates papillary thyroid cancer progression by acting as a CeRNA to affect miR-199a-5p activity (24). TTN-AS1 enhances the metastasis of lung cancer *via* regulating the miR-142-5p/CDK5 axis (25). In this study, we conducted functional enrichment analysis of FAM83H-AS1 in PCa. We show that FAM83H-AS1 is related to the regulation of cell cycle and DNA replication, which is consistent with the above findings. ceRNA network analysis was subsequently conducted and revealed that FAM83H-AS1 may play its role in PCa through the miR-15a/CCNE2 axis. Further validation demonstrates that overexpression of miR-15a remarkably suppressed CCNE2 and FAM83H-AS1 expression. The luciferase reporter assay further shows CCNE2 and FAM83H-AS1 are direct targets of miR-15a. Of note, the rescue assay showed that FAM83H-AS1 promoted cell proliferation and migration of PCa through sponging miR-15a to promote CCNE2 expression in PCa.

miR-15a works as a tumor-suppressive miRNA in gastric cancer (26–28), lung cancer (29, 30), liver cancer (31), and PCa (32, 33). In PCa, the miR-15a/miR-16 cluster was found to suppress tumor invasion by suppressing the TGF-β signaling pathway to inhibit growth through CCND1 and WNT3A (32). In addition, miR-15 and miR-16 are also found to be downregulated in fibroblasts surrounding the prostate tumors. The suppression of miR-15 and miR-16 in fibroblasts enhanced expression of Fgf-2 and Fgfr1 to promote PCa proliferation and migration. Moreover, miR-15a is reported to be reduced in PCa tissues and plasma samples, suggesting it could be a potential biomarker for PCa (34). CCNE2 is a key regulator of cell cycle (35). A recent study shows that CCNE2 is also involved in regulating cancer apoptosis and migration (35, 36). In NSCLC cells, CCNE2 enhances tumor proliferation, invasion, and migration (35). In pancreatic ductal adenocarcinoma, Yang et al. reports that CCNE2 could rescue tigecycline-suppressed cell metastasis.

Several limitations are also included in this study. First, no functional explorations of FAM83H-AS1 in kidney cancer and bladder cancer were performed in this study. In a future study, we will perform more loss- and gain-of-function assays in kidney cancers. Second, the further confirmation of our findings using *in vivo* assays should strengthen the functional importance of FAM83H-AS1.

In conclusion, we, for the first time, reveal a novel AR-regulated lncRNA FAM83H-AS1 promotes PCa progression *via* the miR-15a/CCNE2 axis, suggesting that lnc-FAM83H-AS1 may be a potential biomarker for PCa.

## Data Availability Statement

The original contributions presented in the study are included in the article/**Supplementary Material**. Further inquiries can be directed to the corresponding author.

## Ethics Statement

The studies involving human participants were reviewed and approved by the Ethics Committee of Tongji University. The patients/participants provided their written informed consent to participate in this study.

## Author Contributions

BL and DW designed the study. BL, WZ, and HJ performed the assays and acquired the data. HJ performed the bioinformatics analysis. All authors draft and reviewed the manuscript. All authors contributed to the article and approved the submitted version.

## Conflict of Interest

The authors declare that the research was conducted in the absence of any commercial or financial relationships that could be construed as a potential conflict of interest.
