# Supplementary material for: A Novel Androgen-Induced lncRNA FAM83H-AS1 Promotes Prostate Cancer Progression via the miR-15a/CCNE2 Axis
Source: Front Oncol. 2021 Feb 4;10:620306. doi: 10.3389/fonc.2020.620306 (PMC7890020; doi:10.3389/fonc.2020.620306)
Supplement: Supplementary file 1 [file Image_1.pdf]

Supplementary Material

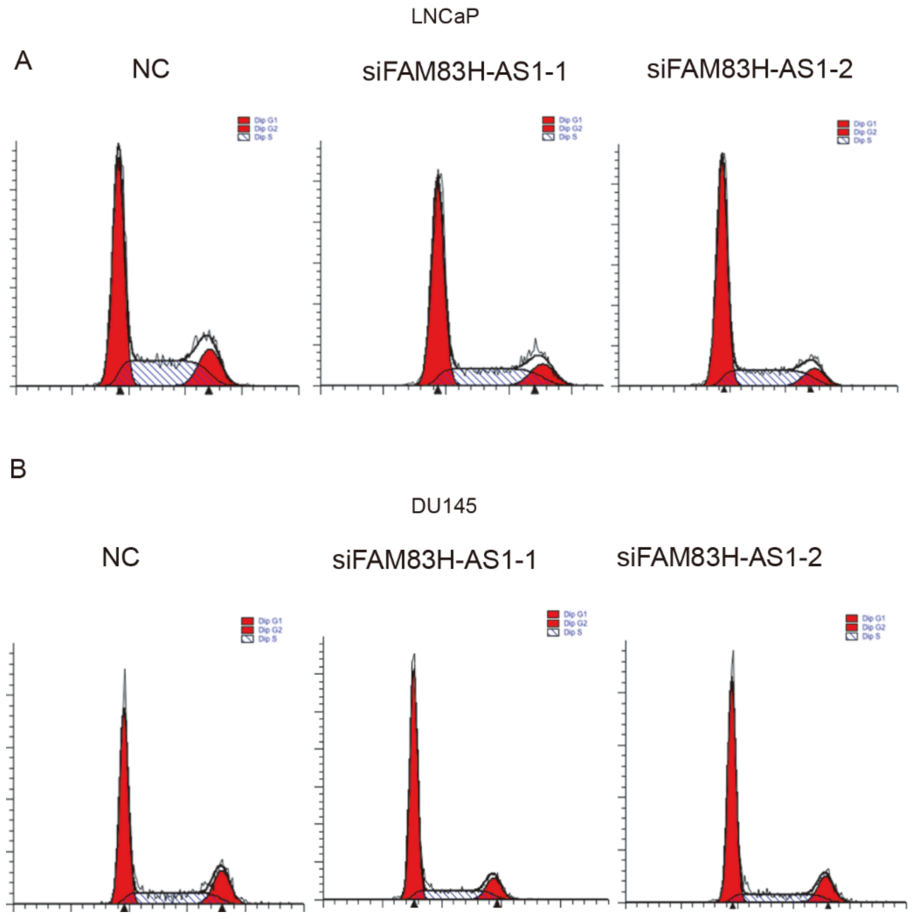

Supplementary Figure 1. Silencing of FAM83H-AS1 suppressed PCa cell cycle. (A-B) flow cytometry assay showed the percentage of G1 phase and S phase after knockdown of FAM83H-AS1 in LNCaP (A) and DU145 (B) cells.
